# Thulium laser enucleation of the prostate vs bipolar transurethral resection of the prostate in moderate-to-large prostates: perioperative and functional outcomes

**DOI:** 10.20452/wiitm.2026.18016

**Published:** 2026-03-04

**Authors:** Samer Al-Rawashdah

**Affiliations:** Department of Special Surgery, Division of Urology, School of Medicine Mutah University Al-Karak Jordan

**Keywords:** benign prostatic hyperplasia, bipolar transurethral resection of the prostate, laser enucleation, thulium laser enucleation of the prostate

## Abstract

**INTRODUCTION:**

Thulium laser enucleation of the prostate (ThuLEP) and bipolar transurethral resection of the prostate (bipolar TURP) are established surgical therapy alternatives that have proved effective in treating benign prostatic hyperplasia (BPH).

**AIM:**

This study aimed to compare perioperative and functional results obtained in patients with moderate-to-large BPH treated with ThuLEP and bipolar TURP.

**MATERIALS AND METHODS:**

This was a retrospective multicenter study of 154 men at a mean (SD) age of 68.4 (7.9) years, who received ThuLEP (n = 78) or bipolar TURP (n = 76) in 2018–2024 in 2 hospitals in Amman, Jordan. The collected data included demographics, comorbidities, perioperative variables, complications, and functional outcomes at 1 and 6 months postoperatively.

**RESULTS:**

ThuLEP was associated with greater mean (SD) tissue removal (61.4 [18.2] vs 44.9 [14.6] g; *P* = 0.001), lower estimated blood loss (120 [65] vs 260 [110] ml; *P* = 0.001), shorter catheterization time (29.4 [9.8] vs 52.1 [14.3] h; *P* = 0.001), and shorter hospital stay (1.6 [0.7] vs 3.1 [1.2] d; *P* <⁠0.001), as compared with TURP. At 6 months, ThuLEP also resulted in a lower mean (SD) International Prostate Symptom Score (6.1 [2.8] vs 8.7 [3.4]; *P* <⁠0.001), better quality of life score (1.1 [0.6] vs 1.8 [0.8];* P* <⁠0.001), and a higher maximum urinary flow rate (22.4 [5.2] vs 17.6 [4.7] ml/s; *P* <⁠0.001). ThuLEP was an independent predictor of a favorable perioperative outcome (adjusted odds ratio, 2.74; 95% CI, 1.32–5.68; *P* = 0.006).

**CONCLUSIONS:**

ThuLEP is safer and more effective in patients with moderate-to-large volume prostates.

## INTRODUCTION

Benign prostatic hyperplasia (BPH) is a typical age-related urological issue in men and one of the main causes of lower urinary tract symptoms, including urinary frequency, urgency, weak stream, nocturia, and incomplete emptying of the bladder. These symptoms can interfere with the quality of life (QoL) to a large extent and, in extreme cases, lead to complications, such as chronic urinary retention, lower urinary tract infections, and urinary bladder and kidney stones. Although it has been observed that pharmacological therapy is effective in the treatment of patients with mild-to-moderate disease, surgical intervention is at times required in men with moderate-to-large prostate volume, or in the cases were conservative management failed.^1^^,^^2^^,^^3^

Transurethral resection of prostate (TURP) has always been viewed as the benchmark surgical intervention for BPH. Bipolar TURP has enhanced the safety spectrum of customary monopolar resection, as it uses saline irrigation to diminish the risk of TUR syndrome and achieve better intraoperative hemostasis.^2^^,^^3^ Bipolar TURP is widely used, well known among most urologists, and still provides a satisfactory degree of symptomatic improvement. Nevertheless, in patients with larger prostates, bipolar TURP may be associated with increased bleeding, longer catheterization time, extended hospitalization, and even failure to remove all affected tissue, which impacts recovery and long-term outcomes.^2^^,^^3^^,^^4^

Over the recent few years, laser-based enucleation methods have gained more acceptance as a valid alternative to resection-based surgery. Thulium laser enucleation of the prostate (ThuLEP) utilizes a continuous-wave thulium laser which enables fine resection at the prostatic capsule and good simultaneous hemostasis.^5^^,^^6^ The enucleation, an anatomical approach to prostate removal, enables near-total clearance of adenomatous tissue, irrespective of prostate size, and is associated reduced bleeding, shorter catheterization time, and faster postoperative healing. Consequently, ThuLEP is a good option for patients with moderate-to-large prostates and those who are at a greater risk of perioperative bleeding.^3^^,^^4^^,^^5^^,^^6^

Numerous comparative studies and systematic reviews conducted in recent years found that ThuLEP yields the same or better functional outcomes than TURP in terms of urinary flow, symptoms, and long-term results.^2^^,^^3^^,^^4^^,^^5^^,^^6^^,^^7^^,^^8^^,^^9^ Its advantages appear more pronounced in the cases involving large prostates, where shortcomings of resection-based methods become more evident.

The existing international literature offers a comparative analysis of laser enucleation vs resection techniques; however, empirical data from the Middle East, particularly those derived from multicenter, real-world clinical settings, are still scarce. Discrepancies in surgical proficiency, learning trajectories, patient demographics, procedural volumes, and overall health care infrastructure can have marked effects on postoperative and functional outcomes, potentially limiting the extrapolability of surgical results obtained in highly controlled experimental trials. While ThuLEP has been increasingly used the Middle East—particularly in Jordan—comparative datasets within the region are still very limited. Consequently, region-specific evidence is essential in determining whether the benefits found in published international studies are replicable in clinical practice.

## AIM

The principal aim of this research was to compare the safety of perioperative processes, effectiveness, as well as short- and mid-term functional outcomes among patients with moderate-to-large BPH who underwent ThuLEP and TURP in Amman, Jordan, in major endourology centers.

## MATERIALS AND METHODS

### Study design

This research used a retrospective chart review approach in order to compare perioperative and functional outcomes of ThuLEP vs bipolar TURP in treating patients with moderate-to-large BPH. The retrospective method has been selected due to the possibility of evaluating real-life outcomes in several centers. All available medical records were accessed without direct contact with the patients.

### Study setting

The study was conducted in 2 hospitals in Amman, Jordan (Istiqlal Hospital and Saudi Hospital), specializing in ThuLEP and bipolar TURP procedures. The procedures included in the study were carried out in 2018–2024.

### Study population and sample size

The analysis included 154 patients who underwent BPH surgery within the study period. The population was divided according to the surgical technique used: ThuLEP (n = 78) and bipolar TURP (n = 76). The inclusion criteria comprised: 1) age of 50 years or older; 2) established diagnosis of BPH; 3) prostate volume of 40 ml or less on transrectal ultrasound (TRUS); 4) undergoing ThuLEP or bipolar TURP; and 5) availability of complete perioperative data with minimum 6-month follow-up. The exclusion criteria encompassed: 1) a history of prostate surgery; 2) known urethral stricture disease; 3) neurogenic bladder dysfunction; 4) active urinary tract infection; 5) conversion to another type of surgery during the procedure; and 6) incomplete medical records.

The participants were grouped based on current clinical practice, surgeon experience, equipment availability, and individual clinical patient characteristics. Patient allocation was protocol-driven, as the study was retrospective in nature.

### Procedures

All surgeries were performed by qualified urologists, trained in either laser enucleation or bipolar resection. ThuLEP was performed through anatomical enucleation at the surgical capsule with a continuous-wave thulium laser, and subsequent enucleated tissue morcellation. Bipolar TURP was carried out with the assistance of bipolar energy that was used to gradually resect the prostate tissue in saline. Perioperative management, such as anesthesia, irrigation, catheter management, and postsurgical care were based on institutional standards. After surgery, the patients were followed for 6 months.^8^^,^^9^^,^^10^^,^^11^^,^^12^

### Study instrument

For the purposes of this study, a structured, comprehensive data collection sheet was designed to standardize the information obtained from the participating centers. The tool consisted of 6 parts. The first part contained demographic information and details of the surgical procedure, including patient age, body mass index (BMI), type of procedure, and surgery date. The second part was devoted to comorbidities, including hypertension, diabetes mellitus, and cardiovascular disease, to consider baseline clinical risk profiles. The third section recorded preoperative assessments, which included prostate volume measurements on TRUS, prostate-specific antigen level, validated functional measures of the International Prostate Symptom Score (IPSS), QoL score, maximum urinary flow rate (Q_max_), and postvoid residual urine volume (PVR).^5^^,^^6^^,^^7^ The fourth part involved intraoperative variables regarding efficiency and safety of the surgical procedures, and included operative time, weight of the resected or enucleated tissue, estimated blood loss, irrigation time, catheterization time, and any intraoperative complications. The fifth section contained early postoperative outcomes, which included length of hospital stay (LOS), complications following the surgery according to the Clavien–Dindo scale, necessity for recatheterization, and 30-day readmission. The final part included functional results at follow-up visits performed 1 and 6 months postoperatively, in which the severity of symptoms, QoL, Q_max_, and PVR were measured again. The analysis of the collected data enabled a comparison of the safety, perioperative functionality, and efficacy of ThuLEP and bipolar TURP.^5^^,^^6^^,^^7^

A favorable perioperative outcome was defined as absence of intra- or postoperative complications (Clavien–Dindo grade ≥II), no indications for blood transfusion, and no need for recatheterization at the time of the first hospitalization.

### Data collection procedures

The data were retrieved from electronic medical records, operative, anesthesia, radiology, and TRUS reports, postoperative progress notes, and follow-up reports provided by the participating centers. The data were extracted by 2 trained independent reviewers (MA and OA) using a standardized sheet. All disputes between the reviewers were resolved through discussion and, where necessary, by a senior urologist (MK). Before analysis, all data were anonymized and coded.

QoL was assessed using the IPSS questionnaire, which contains 7 items measuring lower urinary tract symptoms, and each item is assigned a score from 0 to 5, which results in a total score of 0 to 35. Additionally, the IPSS includes a QoL variable which assesses patient satisfaction with the urinary condition, and is scored on a scale from 0 (delighted) to 6 (terrible). The questionnaire was administered routinely as part of urological care during preoperative evaluation and at follow-up visits 1 and 6 months after surgery. All scores were prospectively recorded in the electronic medical records. For the purposes of the current retrospective analysis, the data on the assessment of the QoL were extracted from these routinely collected clinical documents. No additional data collection methods were used.^5^^,^^6^^,^^7^

### Ethics

The study was reviewed and approved by the Ethics Committee of the Faculty of Medicine of the Mutah University (74025). The committee did not approve any corrections to the study protocol. Due to the retrospective nature of the analysis, informed consent was waived according to the institutional ethics protocol. No personal data were used, since all information was anonymized and data collection, storage, and reporting were performed with high level of confidentiality. The research was conducted according to the provisions of the Declaration of Helsinki and the regulations of the corresponding institutions where it was conducted.

### Statistical analysis

Statistical analysis was performed using SPSS Statistics software, version 27 (IBM Corp., Armonk, New York, United States). Continuous variables were assessed for normality using the Shapiro–Wilk test. The variables with normal distribution are represented as means (SD), while those not normally distributed are described as medians with interquartile ranges (IQRs). Between-group comparisons were performed with the independent samples *t* test for normally distributed continuous variables, and the Mann–Whitney test for skewed variables. Within group pre- and postoperative comparisons were made using the paired *t* test or the Wilcoxon signed-rank test, as appropriate. Categorical variables are expressed as frequencies and percentages, and were compared using the χ^2^ test or the Fisher exact test when expected cell counts were small.

A multivariate logistic regression analysis was used to define independent factors associated with good perioperative outcomes. A favorable perioperative outcome was defined as a composite end point with absence of intra- and postoperative complications according to the Clavien–Dindo grade equal to or higher than II, no need for blood transfusion, and no need for recatheterization during initial hospitalization. The variables were incorporated into a regression model based on clinical relevance and precedent literature, and included age, BMI, prostate volume, diabetes mellitus, and surgical technique (ThuLEP vs bipolar TURP), since each may have an effect on a hemorrhagic risk, incidence of complications, and postoperative recovery. All covariates were included in the model at the same time, and adjusted odds ratios (ORs) with 95% CIs were calculated. Significance was determined at a 2-sided *P *value of 0.05.

## RESULTS

A total of 154 patients were included in the analysis, with 78 individuals in the ThuLEP and 76 in the bipolar TURP groups. Baseline demographic and clinical characteristics were comparable between the cohorts. Mean (SD) patient age was 68.4 (7.9) years in the ThuLEP group and 69.1 (8.2) years in the bipolar TURP group (*P* = 0.56). Mean (SD) prostate volume was 82.5 (21.4) vs 79.8 (19.7) ml, respectively (*P* = 0.41). No intergroup differences were observed in BMI, prostate-specific antigen levels, comorbidity burden, or preoperative functional parameters, including mean (SD) IPSS (22.1 [5.4] vs 21.8 [5.1]; *P* = 0.72), QoL score (4.9 [1.1] vs 4.8 [1.1]; *P* = 0.58), Q_max_ (7.4 [2.3] vs 7.6 [2.1] ml/s; *P* = 0.61), and PVR (median [IQR], 165 [120–230] vs 158 [115–220] ml; *P* = 0.47), confirming baseline equivalence between the groups (TABLE 1).

Intraoperative outcomes demonstrated differences between the 2 techniques. Although mean (SD) operative time was longer for ThuLEP than bipolar TURP (96.2 [18.5] vs 88.7 [16.9] min; *P* = 0.01), ThuLEP achieved greater tissue removal volume (61.4 [18.2] vs 44.9 [14.6] g; *P* <⁠0.001). ThuLEP was associated with lower median (IQR) blood loss (110 [80–160] vs 250 [190–320] ml), shorter irrigation time (10 [8–13] vs 18 [154–22] h), and reduced catheterization duration (28 [2–34] vs 50 [42–60] h), as compared with bipolar TURP (all *P* <⁠0.001), indicating superior perioperative stability and faster postoperative recovery with ThuLEP (TABLE 2).

**TABLE 1:** Baseline demographic and preoperative characteristics of the study population

Variable		ThuLEP (n = 78)	Bipolar TURP (n = 76)	*P* value
Age, y		68.4 (7.9)	69.1 (8.2)	0.56
BMI, kg/m²		27.6 (3.8)	27.9 (4.1)	0.64
Prostate volume, ml		82.5 (21.4)	79.8 (19.7)	0.41
PSA, ng/ml		3.9 (1.8)	4.1 (2)	0.53
Comorbidities	Hypertension	42 (53.8)	39 (51.3)	0.76
	Diabetes mellitus	31 (39.7)	29 (38.2)	0.85
	Cardiovascular disease	18 (23.1)	20 (26.3)	0.64
Preoperative IPSS, points		22.1 (5.4)	21.8 (5.1)	0.72
QoL score, points		4.9 (1.1)	4.8 (1)	0.58
Qmax, ml/s		7.4 (2.3)	7.6 (2.1)	0.61
PVR, ml, median (IQR)		165 (120–230)	158 (115–220)	0.47

Data are presented as mean (SD) and number (percentage) unless indicated otherwise.

Abbreviations: BMI, body mass index; IPSS, International Prostate Symptom Score; IQR, interquartile range; Q_max_, maximum urinary flow rate; PSA, prostate‑specific antigen; QoL, quality of life; PVR, postvoid residual urine volume; ThuLEP, thulium laser enucleation of the prostate; TURP, transurethral resection of the prostate

**TABLE 2:** Intra‑ and perioperative outcomes of thulium laser enucleation of the prostate vs bipolar transurethral resection of the prostate

Variable	ThuLEP (n = 78)	Bipolar TURP (n = 76)	*P* value
Operative time, min	96.2 (18.5)	88.7 (16.9)	0.01
Tissue removed, g	61.4 (18.2)	44.9 (14.6)	<0.001
Estimated blood loss, ml	110 (80–160)	250 (190–320)	<0.001
Irrigation time, h	10 (8–13)	18 (15–22)	<0.001
Catheterization time, h	28 (22–34)	50 (42–60)	<0.001

Data are presented as mean (SD) or median (interquartile range).

Abbreviations: (see TABLE 1)

**TABLE 3:** Early postoperative outcomes and complications

Outcome		ThuLEP (n = 78)	Bipolar TURP (n = 76)	*P* value
Length of hospital stay, d		1.6 (0.7)	3.1 (1.2)	<0.001
Complications	Any complication	7 (9)	18 (23.7)	0.01
	Clavien–Dindo I–II	6 (7.7)	13 (17.1)	
	Clavien–Dindo ≥III	1 (1.3)	5 (6.6)	
Recatheterization		4 (5.1)	11 (14.5)	0.04
30-day readmission		2 (2.6)	8 (10.5)	0.03

Data are presented as mean (SD) or number (percentage).

Abbreviations: see (TABLE 1)

Early postoperative recovery was better in the ThuLEP group across multiple parameters. Mean (SD) LOS was shorter following ThuLEP than bipolar TURP (1.6 [0.7] vs 3.1 [1.2] d; *P* <⁠0.001). Overall postoperative complication rates were lower in the ThuLEP cohort than in the bipolar TURP group (9% vs 23.7%; *P* = 0.01). Most complications were minor (Clavien–Dindo grade, I–II); however, higher-grade complications (≥grade III) occurred more frequently after bipolar TURP (6.6% vs 1.3%). Recatheterization was required in 5.1% of the ThuLEP patients, as compared with 14.5% of the individuals in the bipolar TURP group (*P* = 0.04), and 30-day readmission rates were lower following ThuLEP (2.6% vs 10.5%; *P* = 0.03; TABLE 3).

At 1 month postoperatively, both procedures resulted in marked improvements in urinary symptoms and functional parameters; however, the outcomes were superior in the ThuLEP group. Mean (SD) IPSS was lower after ThuLEP than bipolar TURP (8.2 [3.1] vs 10.4 [3.6]; *P* <⁠0.001), with a corresponding improvement in QoL scores (1.5 [0.7] vs 2.2 [0.9]; *P* <⁠0.001). Urinary flow rates were considerably higher following ThuLEP, with mean (SD) Q_max_ of 19.6 (4.8) vs 15.8 (4.2) ml/s (*P* <⁠0.001). Median (IQR) PVR was also lower in the ThuLEP group, as compared with the bipolar TURP cohort (45 [30–70] vs 75 [50–110] ml; *P* <⁠0.001; TABLE 4).

**TABLE 4:** Functional outcomes at 1‑ and 6 ‑month follow ‑up

Parameter		ThuLEP (n = 78)	Bipolar TURP (n = 76)	*P* value
1-month follow-up	IPSS, points	8.2 (3.1)	10.4 (3.6)	<0.001
	QoL score, points	1.5 (0.7)	2.2 (0.9)	<0.001
	Qmax, ml/s	19.6 (4.8)	15.8 (4.2)	<0.001
	PVR, ml	45 (30–70)	75 (50–110)	<0.001
6-month follow-up	IPSS, points	6.1 (2.8)	8.7 (3.4)	<0.001
	QoL score, points	1.1 (0.6)	1.8 (0.8)	<0.001
	Qmax, ml/s	22.4 (5.2)	17.6 (4.7)	<0.001
	PVR, ml	30 (20–50)	65 (40–90)	<0.001

Data are presented as mean (SD) or median (interquartile range).

Abbreviations: see (TABLE 1)

**TABLE 5:** Multivariable analysis of predictors of favorable perioperative outcomes

Predictor	Adjusted odds ratio (95% CI)	P value
ThuLEP vs bipolar TURP	2.74 (1.32–5.68)	0.006
Prostate volume	0.98 (0.96–1.01)	0.19
Age	0.97 (0.94–1.01)	0.14
BMI	0.99 (0.94–1.05)	0.78
Diabetes mellitus	0.88 (0.42–1.84)	0.74

Abbreviations: see (TABLE 1)

Sustained functional improvements were observed at 6-month follow-up in both groups, with consistently superior outcomes following ThuLEP. Mean (SD) IPSS remained lower for ThuLEP, as compared with bipolar TURP (6.1 [2.8] vs 8.7 [3.4]; *P* <⁠0.001), accompanied by better QoL scores (1.1 [0.6] vs 1.8 [0.8]; *P* <⁠0.001). ThuLEP patients achieved higher urinary flow rates, with mean (SD) Q_max_ of 22.4 (5.2) ml/s, in comparison with 17.6 (4.7) ml/s in the bipolar TURP group (*P* <⁠0.001). Median (IQR) PVR was also markedly reduced following ThuLEP (30 [20–50] vs 65 [40–90] ml; *P* <⁠0.001), indicating more effective long-term bladder emptying (TABLE 4).

The multivariable regression analysis identified ThuLEP as an independent predictor of favorable perioperative outcomes after adjusting for age, BMI, prostate volume, and comorbidities. The patients undergoing ThuLEP were considerably more likely to experience improved perioperative outcomes than those treated with bipolar TURP (adjusted OR, 2.74; 95% CI, 1.32–5.68; *P* = 0.006). No associations were observed between perioperative outcomes and prostate volume (*P* = 0.19), age (*P* = 0.14), BMI (*P* = 0.78), or diabetes mellitus (*P* = 0.74; TABLE 5).

## DISCUSSION

This multicenter retrospective study provided clinically relevant and detailed comparisons between ThuLEP and bipolar TURP in the patients with medium-to-large BPH. The results demonstrate that both surgical methods are effective in the reduction of lower urinary tract symptoms and improvement of functional outcomes, and can be used in modern urologic practice.^2^^,^^3^^,^^4^ However, significant differences were noted in perioperative safety, operative efficiency, and postoperative recovery, reflecting the underlying technical differences between resection-based and anatomical enucleation strategies. ThuLEP exhibited a better and more convincing perioperative profile, particularly in terms of hemorrhagic control, catheter indwelling time, and early postoperative recovery, which reflects the inherent benefits of laser enucleation techniques.^1^^,^^2^^,^^3^

The high rate of hemostatic control with ThuLEP may be due to the physical characteristics of the thulium laser, which enables a continuous-wave mode that delivers shallow tissue penetration and, at the same time, allows for tight dissection along the prostatic capsule and effective coagulation. This attribute is of special advantage in patients with larger prostates, where vascularity is increased and the risk of bleeding is naturally higher.^7-8^ The implications of improved perioperative stability with ThuLEP are significant not only for patient safety but also patient comfort and postoperative resource utilization, as reduced blood loss facilitates earlier catheter removal and hospital discharge. These findings coincide with accumulating international evidence supporting laser enucleation as a less risky alternative, as compared with conventional resection, for larger gland volumes.^3^^,^^4^^,^^5^

Operationally, ThuLEP allowed for a more complete anatomic excision of the obstructing prostatic adenoma, which contributed to stronger and more durable functional benefits observed during follow-up. Anatomical enucleation involves entering the natural surgical plane between the adenoma and the capsule, facilitating near-complete tissue clearance regardless of prostate size. The enucleation technique, although associated with a slightly longer operative time, should be interpreted in the context of its technical demands and learning curve rather than reduced efficiency.^5^^,^^6^^,^^7^ Surgeon experience has been shown to decrease operative time, and the initial investment in training may be offset by improved long-term outcomes. In contrast, bipolar TURP is technically familiar and widely practiced; however, it relies on progressive tissue resection, which may be less effective in achieving complete removal of large adenomas, leaving residual obstructive tissue.^9^^,^^10^^,^^11^

The favorable outcomes of ThuLEP were further supported by early postoperative results, including fewer complications and smoother recovery. Lower rates of recatheterization and readmission indicate better early urinary control and recovery of voiding function. Notably, although both procedures demonstrated acceptable safety profiles, the substantially lower complication burden observed after ThuLEP provides valid justification for considering it a minimally-invasive yet comprehensive surgical option. The predominance of minor complications in both groups emphasizes that both methods are safe when performed by experienced surgeons; however, the additional safety margin provided by ThuLEP is particularly valuable in geriatric patients and those with multiple comorbidities.^10^^,^^11^^,^^12^

Both surgical approaches were clinically effective based on functional outcomes at short- and mid-term follow-up, as demonstrated by sustained symptom reduction and improved urinary flow. However, greater and more durable improvements in symptom severity and QoL were achieved in the individuals undergoing ThuLEP. These long-term advantages may be attributed to complete adenoma enucleation, which minimizes residual obstruction and reduces the likelihood of symptom recurrence.^1^^,^^2^^,^^3^ The sustainability of functional outcomes observed with ThuLEP is consistent with international comparative studies and meta-analyses, which similarly report superior long-term symptom control and lower retreatment rates with laser enucleation, in comparison with resection-based techniques. Collectively, these findings suggest an ongoing paradigm shift toward anatomical laser enucleation as the preferred surgical approach for managing moderate-to-large BPH.^4^^,^^5^^,^^6^^,^^7^

In contrast to randomized trials conducted in highly controlled settings, the present study reflects real-world surgical practice of 2 centers that are characterized by differential surgeon experience and patient demographics. This strengthens the external validity of the findings and the reproducibility of the ThuLEP results within the routine clinical practice of the Middle Eastern health care systems.

### Clinical implications

The results of this study can be applied in the selection of the surgical technique for patients diagnosed with moderate-to-large BPH. ThuLEP appears to have a more favorable prospective operating profile, offers faster recovery and long-lasting experimental results, and can be more suitable for patients with large prostate volumes or a high bleeding risk. Although bipolar TURP is effective and widely used, its application may be more adequate for smaller prostate sizes. Implementation of ThuLEP in high-volume centers can help reduce LOS, postoperative complication rates, and improve patient satisfaction.

### Limitations

The study has certain shortcomings to be acknowledged when interpreting the findings. Its retrospective design may have contributed to selection bias and unrestricted confounding. Due its cross-institutional format, this study may have been affected by variations in surgeon experience and institutional procedures, which could have interfered with the results, in particular operative time and comorbidities. Also, the follow-up was only 6 months, which might not reflect long-term stability, relapse, or late complication rates. Lastly, clinical documentation and patient-reported outcomes were the basis of functional outcomes, which may have introduced subjectivity.

### Recommendations for future research

Future studies should use prospective and randomized design to limit bias and increase causal inferences. Longer follow-up is needed to determine long-term functional outcomes, rates of repression, and cost-efficiency. Regional or multinational registry-based research might also be of benefit in the standardization of outcome reporting and optimization of patient selection for a particular type of surgery.

## CONCLUSIONS

ThuLEP and TURP are safe and effective procedures for BPH. Nonetheless, in patients with moderate-to-large prostate volumes, ThuLEP has clear advantages in terms of perioperative safety, recovery pattern, and long-term functional improvements. ThuLEP should be the preferred surgical option in well-equipped centers.

## JOURNAL INFORMATION

*Videosurgery and Other Miniinvasive Techniques *is an official journal of the Videosurgery Foundation.
